# Prevalence of erectile dysfunction in male survivors of cancer: a systematic review and meta-analysis of cross-sectional studies

**DOI:** 10.3399/bjgp20X714197

**Published:** 2021-04-30

**Authors:** Damiano Pizzol, Tao Xiao, Lee Smith, Guillermo F López Sánchez, Andrea Garolla, Christopher Parris, Yvonne Barnett, Petre Cristian Ilie, Pinar Soysal, Jae Il Shin, Mark A Tully, Lin Yang, Nicola Veronese, Igor Grabovac

**Affiliations:** Italian Agency for Development Cooperation, Khartoum, Sudan.; College of Mathematics and Statistics, Shenzhen University, Shenzhen, China.; Cambridge Centre for Sport and Exercise Sciences, Anglia Ruskin University, Cambridge, UK.; Faculty of Sport Sciences, University of Murcia, Spain.; Unit of Andrology and Reproductive Medicine, Department of Medicine, University of Padova, Padua, Italy.; Biomedical Research Group, Faculty of Science and Engineering, Anglia Ruskin University, Cambridge, UK.; Biomedical Research Group, Faculty of Science and Engineering, Anglia Ruskin University, Cambridge, UK.; Queen Elizabeth Hospital King’s Lynn NHS Foundation Trust, King’s Lynn, UK.; Department of Geriatric Medicine, Bezmialem Vakif University, Istanbul, Turkey.; Department of Paediatrics, Yonsei University College of Medicine, Seoul, Korea.; School of Health Sciences, Institute of Mental Health Sciences, Ulster University, Newtownabbey, Northern Ireland.; Department of Cancer Epidemiology and Prevention Research, Alberta Health Services, Holy Cross Centre, Canada.; Neuroscience Institute, Aging Branch, National Research Council, Padua, Italy.; Department of Social and Preventive Medicine, Centre for Public Health, Medical University of Vienna, Vienna, Austria.

**Keywords:** erectile dysfunction, male cancer, meta-analysis, systematic review, prevalence

## Abstract

**Background:**

Prevalence of erectile dysfunction (ED) in male survivors of cancer across cancer types has not been systematically analysed.

**Aim:**

To estimate the prevalence of ED in all types of cancer and identify characteristics associated with ED in survivors of cancer.

**Design and setting:**

Systematic review and meta-analysis (MA) of cross-sectional studies.

**Method:**

MEDLINE, CINAHL, PsycINFO, and EMBASE were searched, targeting reports published from inception to 1 February 2020. All retrospective or prospective studies reporting prevalence of ED in male patients with cancer and using a validated tool for detection of ED were included. A random-effects MA model was used to pool prevalence of ED as absolute estimates at three different stages, that is, ‘healthy’, ‘at diagnosis’, and ‘after treatment’. A univariate MA regression including the three-level group variable as the only independent variable was used to assess the difference in ED prevalence across the three groups. Further MAs were conducted for studies involving patients at diagnosis and after treatment, and statistical inferences were made with setting for multiple testing controlling for a false discovery rate (FDR) <0.05.

**Results:**

In total, 1301 studies were assessed for inclusion. Of these, 141 were potentially eligible and subsequently scrutinised in full text. Finally, 43 studies were included with a total of 13 148 participants. Overall, pooled data of the included studies showed an ED prevalence of 40.72% (95% confidence interval [CI] = 31.80 to 50.29) in patients with cancer, with prevalences of 28.60% (95% CI = 12.10 to 53.83) at time of diagnosis and 42.70% (95% CI = 32.97 to 53.03) after treatment, with significant difference between these two stages and across cancer locations, controlling for an FDR <0.05.

**Conclusion:**

Erectile dysfunction was particularly high in male survivors of cancer and was associated with cancer treatment, cancer site, and age.

## INTRODUCTION

Cancers located in the pelvic region represent >25% of all newly diagnosed cancers worldwide in males.^1^ This localisation of cancer has also been associated with long-term severe sexual dysfunction in at least half of all patients.^2^ Erectile dysfunction (ED), the inability to obtain or maintain an erection that allows for sexual intercourse, is one of the most distressing consequences of cancer diagnosis and treatment in males.^3^

Erectile dysfunction has a complex aetiology influenced by cancer in both direct and indirect ways. Males diagnosed with prostate cancer, the second most common type of cancer (except for non-melanoma skin cancer) in males,^1^ are expected to have the same risk factors (cardiovascular disease and metabolic disorders) for ED when compared with cancer-free age-matched males. However, risks for ED are increased given a higher incidence of lower urinary tract symptoms and psychological distress in males with prostate cancer.^4^^,^^5^ Indirect pathways, mostly associated with cancer treatment modalities (surgery, chemo- and radiotherapy, and hormone treatment) seem to be the most common causes.^6^^,^^7^

Moreover, few males are able to achieve a normal erection following pelvic surgery, with studies noting that, even in males with excellent baseline erections, <25% retained or recovered the erection quality as before treatment. Pelvic surgeries most associated with ED are radical prostatectomy, radical cystectomy, and low anterior or abdominoperineal resections.^8^ Furthermore, the results from a 12-year follow-up study showed that 84% and 80%, respectively, of males with prostate cancer who had radical prostatectomy or were under active surveillance reported ED, compared with 43% in the matched control group.^9^ Similar results have been reported for males who had treatment for other types of pelvic cancer, such as anal, rectal, or bladder cancer.^10^^–^^15^ However, it is noteworthy that ED is not only prevalent in males with pelvic cancers but may also be the result of intensive chemo- or radiotherapy, causing hypogonadism or pelvic nerve damage. Studies have shown ED also after lung cancer, haematological malignancies, and head and neck tumours.^16^^–^^18^

**Table:** How this fits in

In male survivors of cancer, normal sexual function may be disturbed owing to the occurrence of erectile dysfunction (ED). The present systematic review and meta-analysis reports 40.72% prevalence of ED in survivors of cancer, with the prevalence being somewhat higher (42.70%) in studies that focused on reporting prevalence after cancer treatment. The reasons for high occurrence of ED in male survivors of cancer is multimodal and includes a variety of factors, such as psychological and physical ones. Clinicians should be aware that ED has a large effect on the quality of life and mental health of male survivors of cancer.

Sexuality and intimacy are important aspects of quality of life and may also reduce some of the psychosocial distress associated with the cancer diagnosis. In this light it has been reported that maintaining normal sexual function in males with cancer can be important to help relieve suffering.^19^^,^^20^ Given the growing incidence of cancer globally and new therapeutic modalities that are prolonging life expectancy in survivors of cancer, questions of quality of life post-diagnosis and treatment are increasingly relevant. However, studies on ED in survivors of cancer are rare, and mostly focused on cancer localisations in the pelvic region, making prevalence estimates of ED in survivors of cancer rare. Providing pooled estimates of ED prevalence as well as its associations should provide important information not only on the scale of the issue but also help clinicians working with survivors of cancer to easily identify patients who are at risk of ED, and provide comprehensive cancer care associated with long-term quality of life.

Therefore, the aim of this systematic review and meta-analysis (MA) was to examine the available studies and provide pooled estimates for ED prevalence in relation to all cancer sites and identify characteristics associated with ED in survivors of cancer. To the authors’ knowledge, this is the first study of its kind.

## METHOD

### Search strategy

Four electronic databases, MEDLINE, CINAHL, PsycINFO, and EMBASE, were searched, targeting reports published from database inception to 1 February 2020. Terms included in the search strategy are reported in Supplementary Table S1.

The references of retrieved articles, together with the proceedings of relevant conferences, were hand-searched in order to identify other potentially eligible studies for inclusion that were missed by the initial search, or any unpublished data.

The literature search, assessment of inclusion and exclusion criteria, quality of studies, and extraction of data were independently undertaken and verified by the first and second authors. The results were then compared and, in case of discrepancies, a consensus was reached with the involvement of the third author. There was no language restriction.

### Type of studies, inclusion and exclusion criteria

All retrospective or prospective studies reporting the prevalence of ED in male patients with cancer and using a validated tool for ED detection, for example, the International Index of Erectile Function (IIEF-5), were included in this review. Studies that did not meet the inclusion criteria were excluded.

### Types of outcome measures

All outcomes were defined before conducting the literature search. The primary outcome was the prevalence of ED across relevant cancer treatment stages, that is, ‘healthy’, ‘at diagnosis’, and ‘after treatment’.

### Data extraction and statistical analyses

Descriptive tables for population and study characteristics were generated for all included studies. The first author, publication year, country of investigators, sample size, age, method of assessment of ED, and cancer type and site were recorded. Furthermore, number of patients with ED among case and control groups, body mass index, hormonal levels, smoking status, and presence of hypertension, diabetes, dyslipidaemia, and cardiovascular diseases were recorded. All statistical analyses based on these data were performed using R (version 3.6.1).

For the included studies at the three different stages, that is, ‘healthy’, ‘at diagnosis’, and ‘after treatment’, a random-effects MA model with the between-study heterogeneity parameter estimated by DerSimonian–Laird (DL) method^21^ was used to pool the prevalence of ED as absolute estimates (%) with their 95% confidence intervals (CIs) for each stage. A univariate MA regression including the three-level group variable for healthy/at diagnosis/after treatment stages as the only independent variable was used to assess the difference in ED prevalence across the three stages. A scatter plot with point and CI estimates of prevalence of ED across three different groups of patients is illustrated. Publication bias was assessed by a visual inspection of funnel plots and calculating the Egger bias test.^22^ The authors planned to apply the trim and fill analysis^23^ for overcoming possible publication bias (*P*<0.10).

Further MA were conducted for the 40 studies only involving patients at diagnosis and after treatment, that is, excluding healthy control. Graphical comparisons of the prevalence of ED across these two stages of cancer treatment were given by a classic forest plot. Heterogeneity across these 40 studies involving the two cancer treatment stages was assessed by the *I*^2^ metric and taking, as measure of high heterogeneity, an *I*^2^ >50% or *P*<0.05 for testing the χ^2^-distributed Q statistic for between-studies heterogeneity (a high value of Q would result in a high value of *I*^2^ since *I*^2^ = [Q-K+1]/Q where K is the number of studies).^24^ In case of high ED-prevalence heterogeneity and having at least 10 studies for the outcome, the authors used stage, continent, mean age, age range, age standard deviation, method of ED assessment, cancer site, proportion of patients that underwent radiotherapy, proportion of patients with diabetes, and proportion of patients that underwent chemotherapy as possible predictors for MA regression analyses. The plots of study count distribution for each of the above moderators across their observed values are given. A univariate MA regression model for each moderator was fitted. The stage predictor as well as the significant moderators screened out by these univariate MA regression analyses were used as potential predictors to fit a multiple MA regression with manual variable selection procedure applied. The conclusions by the final multiple MA regression model were drawn with multiple testing concern by controlling for a false discovery rate (FDR).^25^ Back-transformed estimated prevalence values of ED with 95% CI for studies with different levels of predictor variables in the final multiple MA regression model are given.

For all MA regression, the authors applied the logit transformation to the observed prevalence across primary studies to make the transformed prevalence follow a normal distribution, and the MA regression analysis was based on the transformed scale.

### Assessment of study quality

Study quality was assessed by two investigators (first and third authors) using the Newcastle–Ottawa Scale (NOS).^26^^,^^27^ This scale has been adapted from the Newcastle–Ottawa Quality Assessment Scale for cohort studies to perform a quality assessment of cross-sectional studies for the systematic review. A third reviewer was available for mediation (thirteenth author). The NOS assigns a maximum of 9 points based on three quality parameters: selection, comparability, and outcome.

## RESULTS

The electronic search yielded 1301 studies, after de-duplication, that were assessed for inclusion in the review. Of these, 141 were potentially eligible and subsequently scrutinised in full text (see Supplementary Figure S1).

### Excluded studies

Among the relevant studies, 98 failed to meet the inclusion criteria and were excluded from this review. Of these, 37 used no validated tools for ED assessment, 36 had no useful data on ED prevalence, 18 were longitudinal studies, four had no data on the association between ED and cancer, and three were double publications.

### Included studies

The 43 studies that were included, 36 prospective and 7 retrospective, contained a total of 13 148 participants.^28^^–^^59^ The majority of the studies (*n* = 25) were conducted in Europe, with those remaining in North America (*n* = 6), Asia (*n* = 6), the Middle East (*n* = 5), and Oceania (*n* = 1). The most affected cancer sites were prostate and rectum (*n* = 12 studies, respectively), testis (*n* = 6), haematological (*n* = 5), multiple (*n* = 3), colorectal (*n* = 2), and penis, colon, and anus (*n* = 1 each).

According to NOS, the median quality of the studies was 4.97 (range 3–7), indicating an overall good quality of studies (Supplementary Table S2). In particular, the majority of the studies (*n* = 18) scored 5, followed by 11 studies with 4. Only three studies scored 3 while six and five studies scored 6 and 7, respectively.

### Meta-analysis on prevalence of ED across three stages: healthy, at diagnosis, and after treatment

Distribution of study counts and the corresponding pooled prevalence of ED at the three different stages are shown in Table 1. The pooled prevalence of ED at the ‘after treatment’ stage was statistically significantly different from that of ‘healthy control’ by the univariate MA regression analysis with dummy variables for stage (*P* = 0.0322).

**Table 1.** Study counts and pooled prevalence of ED across three stages

**Counts/prevalence**	**Stage**			
	**Healthy control**	**At diagnosis**	**After treatment**	**Total**
Study count, *n*	3	5	35	43
Pooled number of patients with ED, *n*	250	782	2794	3826
Pooled sample size, *n*	1240	2403	9505	13 148
Pooled prevalence (95% CI)	0.1370 (0.0394 to 0.3808)	0.2861 (0.1229 to 0.5340)	0.4269^a^ (0.3311 to 0.5286)	—

a *The ED prevalence among patients with cancer ‘after treatment’ was statistically significantly different from that of ‘healthy control’ at level 0.05* *(P = 0.0322; this can also be seen by the fact that the point estimate of ED prevalence for ‘healthy control’, 0.1370, is not included in the 95% CI of ED prevalence for patients with cancer after treatment). CI = confidence interval. ED = erectile dysfunction.*

**Figure 1.**
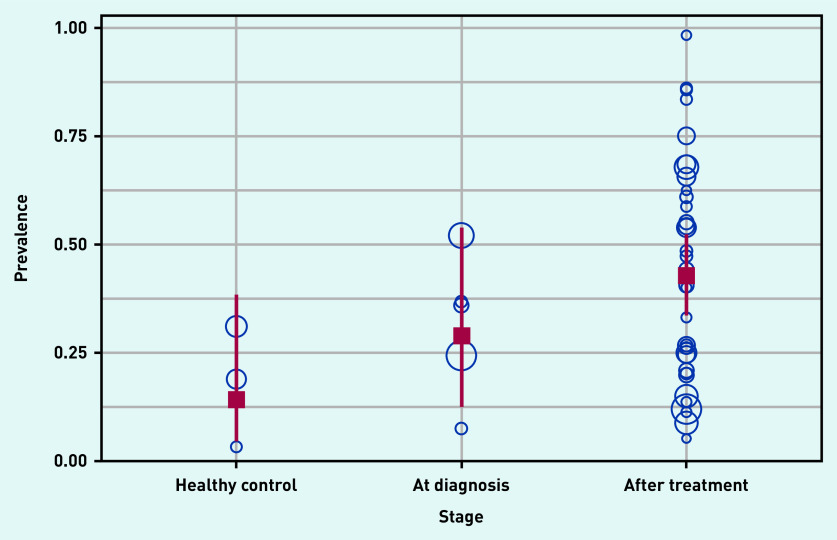
***Comparisons of prevalence of ED among patients with cancer and healthy control. Pooled prevalence of ED represented by red square and the corresponding CIs shown in red extending line (blue circles are centred at the prevalence of ED reported in each of the included primary studies with circle size proportional to sample size of each primary study). ED = erectile dysfunction.***

The pooled prevalence of these three groups is illustrated in Figure 1 to compare prevalence of ED among patients in the two cancer treatment stages with that of ‘healthy control’ individuals.

**Figure 2.**
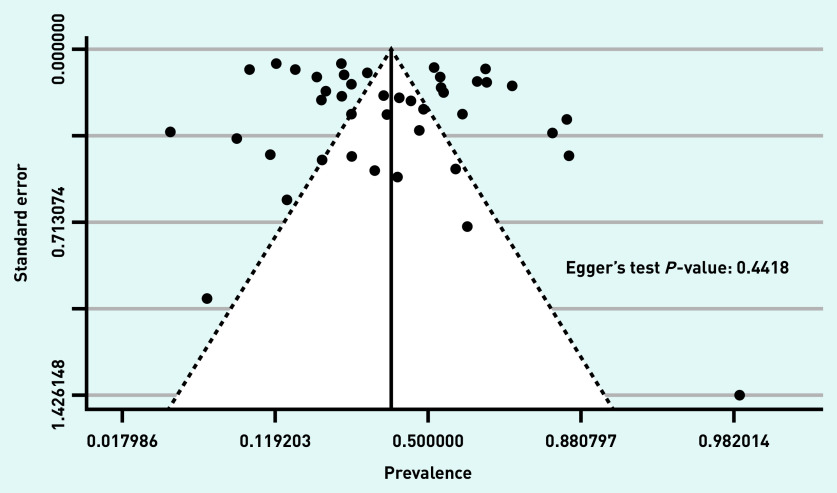
***Funnel plot.***

Small study effect (including publication bias) was not found among the included studies and the trim and fill analysis did not modify the results. Figure 2 shows the funnel plot, with non-significant Egger’s test result for funnel plot asymmetry (*P* = 0.4418).

### Meta-analysis on prevalence of ED across the two cancer treatment stages: at diagnosis and after treatment

**Figure 3.**
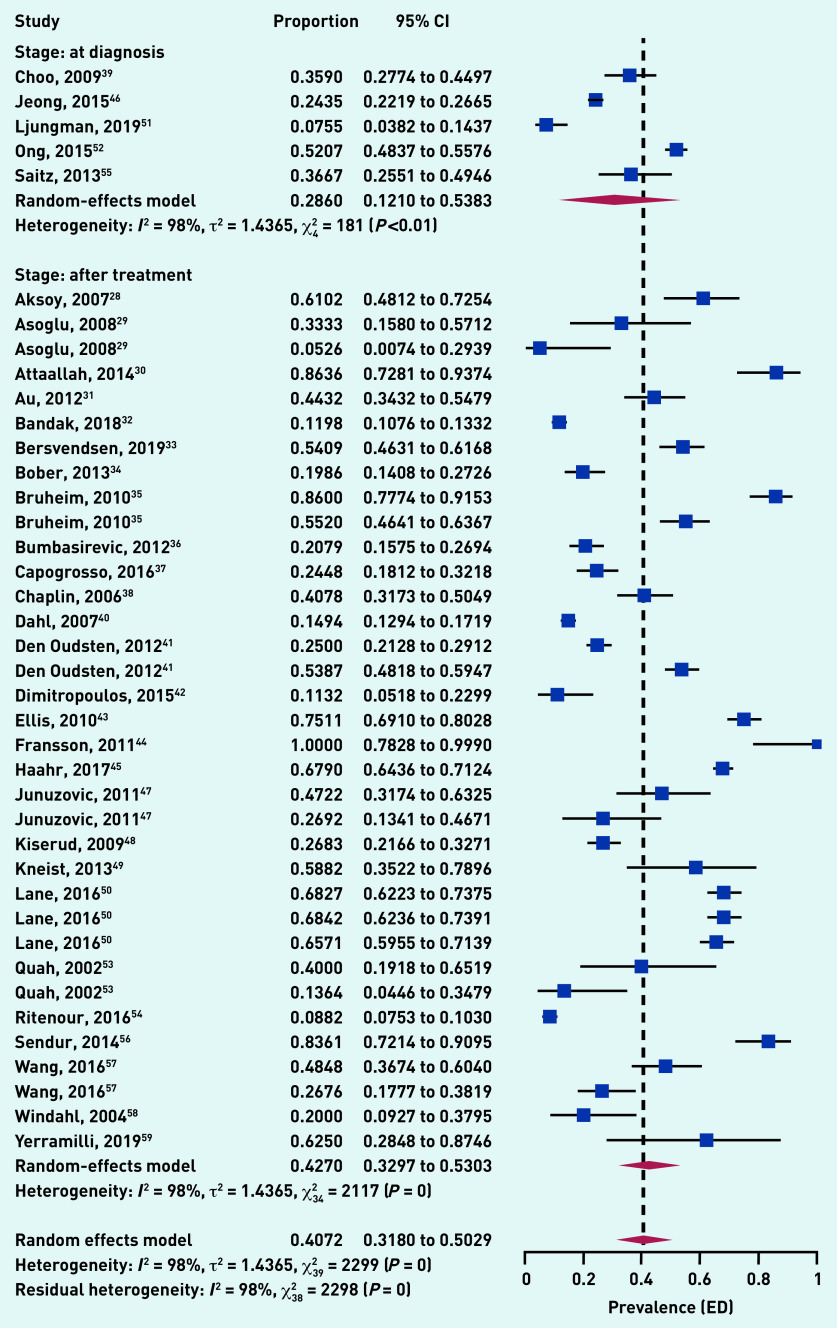
***Prevalence of ED among patients with cancer. ED = erectile dysfunction.***

Pooling data of the 40 studies of patients with cancer only, that is, excluding three studies of healthy controls, an overall prevalence of 40.72% (95% CI = 31.80% to 50.29%) was found, with a prevalence of 28.60% (95% CI = 12.10% to 53.83%) at time of diagnosis and 42.70% (95% CI = 32.97% to 53.03%) after treatment, across cancer locations. A high degree of overall heterogeneity (*I*^2^ = 98%; *P*<0.001) was found. Figure 3 shows the prevalence of ED among patients with cancer.

To locate the potential predictors that account for the very high heterogeneity of ED prevalence among all the primary studies involving patients with cancer in the two treatment stages, MA regression analyses were conducted, with 10 predictors used. Distribution plots of study counts for each of the 10 possible predictors are shown in Supplementary Figure S2. Study counts of these 10 possible predictors for the ED prevalence among patients with cancer and *P*-values for continuous predictor (or smallest *P*-value for the dummy variables of categorical predictor) in the univariate MA regression analysis are shown in Table 2. The results by the univariate MA regression showed that ‘mean age’ and ‘cancer site’ variables were significantly associated with the ED prevalence.

**Table 2.** Study counts of the 10 possible predictors for ED prevalence and *P*-value for the uni-predictor (or smallest *P*-value for the uni-predictor dummy variables) in the univariate MA regression analysis

**Predictor**	**Study count,** ***n***	**Coefficient estimate**	**95% CI**	***P*-value**
Stage	40	0.6210	−0.5253 to 1.7673	0.2883
Continent	40	1.2655	−0.3051 to 2.8362	0.1143
Mean age	40	0.0503	0.0243 to 0.0762	0.0002^a^
Age range	40	0.0057	−0.0210 to 0.0325	0.6739
ED assessment method	40	1.5236	−0.1845 to 3.2316	0.0804
Cancer site	40	−1.8135	−2.5841 to −1.0429	<0.0001^b^
Age standard deviation	17	−0.0078	−0.0733 to 0.0576	0.8144
Proportion of patients who underwent radiotherapy	15	1.0584	−1.0225 to 3.1393	0.3188
Proportion of patients with diabetes	12	−3.6361	−19.1360 to 11.8639	0.6457
Proportion of patients who underwent chemotherapy	12	0.1348	−1.9925 to 2.2621	0.9012

a *Significance code controlling for type I error rate* <*0.05 and* >*0.01.*

b *Significance code controlling for type I error rate* <*0.0001. CI = confidence interval. ED = erectile dysfunction. MA = meta-analysis.*

After a manual variable selection accounting for the multicollinearities of the predictors, a parsimonious MA regression model was built to predict the highly heterogeneous ED prevalence. Regression coefficient estimates of this prediction model are shown in Table 3. This model only included two predictors: stage and cancer site. Since both of these predictors are categorical variables, dummy variables were created to represent them. The reference level for stage was selected as ‘at diagnosis’, and the reference level for cancer site was selected as ‘prostate’, since prostate cancer has the highest count (*n* = 12) in the collected primary study data (this count is the same as for rectum cancer) and prostate cancer is a common cancer in urology. Both predictors are significantly controlling for an FDR <0.05 in this MA regression model, indicating that the ED prevalence estimates reported by primary studies were significantly associated with factors of stage and cancer site. The interpretations of those significant regression coefficients are given as follows: study-reported odds of ED at after-treatment stage are estimated to be 2.4823 (exponential of 0.9092) times that of at-diagnosis stage controlling for other covariates (95% CI = 1.3054 to 4.7204; adjusted *P*-value controlling for FDR = 0.0204); study-reported odds of ED for patients with colon cancer are estimated to be 0.2300 (exponential of −1.4697) times that of patients with prostate cancer controlling for other covariates (95% CI = 0.0697 to 0.7587; adjusted *P*-value controlling for FDR = 0.0434); study-reported odds of ED for patients with lymphoma cancer are estimated to be 0.2530 (exponential of −1.3744) times that of patients with prostate cancer controlling for other covariates (95% CI = 0.0756 to 0.8470; adjusted *P*-value controlling for FDR = 0.0473); study-reported odds of ED for patients with multiple cancers are estimated to be 0.1041 (exponential of −2.2625) times that of patients with prostate cancer controlling for other covariates (95% CI = 0.0419 to 0.2586; adjusted *P*-value controlling for FDR <0.0001); study-reported odds of ED for patients with penis cancer are estimated to be 0.1725 (exponential of −1.7574) times that of patients with prostate cancer controlling for other covariates (95% CI = 0.0394 to 0.7553; adjusted *P*-value controlling for FDR = 0.0433); study-reported odds of ED for patients with testis cancer are estimated to be 0.1353 (exponential of −2.0001) times that of patients with prostate cancer controlling for other covariates (95% CI = 0.0730 to 0.2508; adjusted *P*-value controlling for FDR <0.0001).

**Table 3.** Prediction model for highly heterogeneous ED prevalence

**Regression coefficients**	**Estimate**	**Standard error**	**z**	***P*-value**	**Adjusted** ***P*-value**
Intercept	−0.5380	0.2720	−1.9778	0.0479	0.0659

Stage: after treatment	0.9092	0.3279	2.7726	0.0056	0.0204^a^

**Cancer site**					
Colon	−1.4697	0.6089	−2.4136	0.0158	0.0434^a^
Colorectal	0.9628	0.4856	1.9830	0.0474	0.0744
Haematological	−0.0742	0.4759	−0.1560	0.8761	0.9637
Lymphoma	−1.3744	0.6165	−2.2293	0.0258	0.0473^a^
Multiple	−2.2625	0.4643	−4.8726	<0.0001	<0.0001^b^
Penis	−1.7574	0.7535	−2.3324	0.0197	0.0433^a^
Anus	0.1397	0.9449	0.1478	0.8825	0.8825
Rectum	−0.3761	0.2919	−1.2888	0.1975	0.2414
Testis	−2.0001	0.3148	−6.3533	<0.0001	<0.0001^b^

a *Significance code controlling for false discovery rate (FDR)* <*0.05 and* >*0.01.*

b *Significance code controlling for FDR* <*0.0001. ED = erectile dysfunction.*

The *R*^2^ value of this MA regression is as high as 75.70%, indicating that this MA regression model already accounts for 75.70% heterogeneity of ED prevalence reported by the 40 studies involving patients with cancer (data not shown). The back-transformed estimated ED prevalence values for studies with patients of different cancers at the two stages by this MA regression are shown in Table 4.

**Table 4.** Back-transformed estimated ED prevalence values for studies with patients of different cancers at two stages by the predictive MA regression model

**Cancer site**	**Prevalence at diagnosis, % (95% CI)**	**Prevalence at treatment, % (95% CI)**
Prostate	59.2 (48.7 to 68.9)	78.3 (58.2 to 90.3)
Colon	25.0 (9.8 to 50.4)	45.3 (18.6 to 75.0)
Colorectal	79.1 (61.8 to 89.9)	90.4 (76.4 to 96.5)
Haematological	57.4 (37.0 to 75.5)	77.0 (53.9 to 90.5)
Lymphoma	26.8 (10.6 to 53.2)	47.6 (19.9 to 77.0)
Multiple	13.1 (6.3 to 25.2)	27.2 (11.8 to 51.2)
Penis	20.0 (5.7 to 50.7)	38.3 (11.6 to 74.6)
Anus	62.5 (21.6 to 91.0)	80.5 (37.9 to 96.6)
Rectum	49.9 (40.4 to 59.4)	71.2 (53.9 to 83.9)
Testis	16.4 (10.7 to 24.3)	32.7 (16.8 to 53.9)

CI = confidence interval. MA = meta-analysis.

## DISCUSSION

### Summary

In the present systematic review the search yielded 1301 individual studies, of which 43 studies with a total of 13 148 participants were included in the analysis. The study provides pooled estimates for ED in survivors of cancer across all cancer sites, providing this kind of synthesised data for the first time. Overall, pooled data of the included studies showed an ED prevalence of 40.72% (95% CI = 31.80% to 50.29%) in patients with cancer, with a prevalence of 28.60% (95% CI = 12.10% to 53.83%) at time of diagnosis and 42.70% (95% CI = 32.97% to 53.03%) after treatment, across cancer locations.

Erectile dysfunction was particularly high in male survivors of cancer and was found to be associated with cancer treatment, cancer site, and age.

### Strengths and limitations

This systematic review and MA provide a comprehensive overview of evidence on ED prevalence in survivors of cancer in general, with studies using validated self-reported methods.

Limitations of the present analysis include the inherent limitations from the included studies. Study populations were on average aged >60 years, which may have contributed to the prevalence as ED risks increase with age. This is similar to the over-representation of cancer sites in the pelvic area. Again, because of the small number of primary studies that provided complete clinical and biological (for example, serum testosterone or oestradiol levels) features of the participants, the authors were not able to run some meta-regression analyses using well-known independent risk factors for ED (such as dyslipidaemia, hypertension, diabetes mellitus, and depression) as moderators of the present findings. Lastly, the results pertaining to survivors of cancer with multiple cancer sites need to be taken with caution given that there were only three primary studies that were included in the analysis.

### Comparison with existing literature

Meta-analyses of studies reporting ED prevalence levels in healthy males are rare and mostly focus on samples of Asian males. These studies report that ED prevalence in individual studies has been reported from 2% to 82%, differing among age groups and how ED has been assessed. Generally, lowest reports have been found among younger males aged between 20 and 29 years at 15.1% (99% CI = 12.2% to 18.1%), while the highest have been found in the groups aged ≥60 years at 70.0% (99% CI = 62.3% to 77.7%).^60^ Studies have noted that self-reporting leads to lower estimates than measuring by a standardised questionnaire.^61^^,^^62^ Overall pooled estimate for ED prevalence has been reported at 49.69% (95% CI = 39.29% to 60.10%) for Chinese samples.^63^

Most studies included in the presented MA focused on cancers located in the pelvic region (prostate and rectum) and testis, where the effects would be expected to be strongest given the possible neurovascular damage associated with treatment. Androgen-deprivation therapy (ADT), which is used in prostate cancer management, leads to ED in most males who did not have dysfunction before therapy.^64^^–^^66^ Various chemotherapeutic agents may induce microangiopathy and vascular insufficiency in the *corpus cavernosum* of the penis as well as neurotoxicity that may result in ED.^67^ In a study of >260 males on platinum-based chemotherapy, 40% were reported to have ED on standardised questionnaires, which corresponds to pooled data in the present analysis.^68^ Erectile dysfunction is also a common finding after radiation therapy for prostate cancer with varying incidence reported in studies depending on dose, technique, associated treatments, and time post-treatment, with brachytherapy showing lower rates of ED compared with external-beam radiation therapy in some studies.^69^^,^^70^

Surgical cancer treatment in the pelvic area may also lead to post-operative sexual dysfunction, depending both on the surgical techniques and methods used in assessing ED post-operatively. In one study, >90% of patients who had radical prostatectomy reported lower scores on the IIEF-5 than before surgery,^71^ with an Italian-based study reporting that reaching perioperative levels does not equal patient satisfaction, with little over 25% of patients who reported preoperative scores being satisfied. Only males who achieved scores >22, as measured by the IIEF-5, and who returned to the same levels post-operatively were also satisfied with their sexual function.^72^ Similarly, 86% of males who had radical cystectomy were not able to achieve vaginal penetration^73^ and studies report between 10% and 50% of males having sexual dysfunction following colorectal surgery, where the proposed mechanism may lie in the injury to the hypogastric plexus.^43^^,^^74^ In survivors of testicular cancer, a study measuring blood flow and erectile haemodynamic using duplex ultrasonography reported that 12 months after treatment there were no differences between males with or without hypogonadism, suggesting hyperadrenergic mediated causes of ED.^75^

Sexual function may be influenced by systemic chemo- or radiotherapy, as well as by psychological factors, such as depression, anxiety, low self-esteem, or issues with body image, which are known conditions in all patients with cancer and survivors, regardless of the primary cancer site.^18^^,^^76^^–^^81^ However, very few studies examine the effects of cancer sites outside of the pelvic area on overall sexual function or ED, specifically, in males. An MA on sexual functioning in male lymphoma survivors reported prevalence of sexual dysfunction between 20% and 54%.^82^ Anecdotal evidence also suggests similar prevalence in patients with lung cancer. In fact, though this is the most prevalent cancer in males globally, to the authors’ knowledge there is still no research on sexual function in male patients with lung cancer or survivors, as most of the focus is on short-term survival rather than post-treatment quality of life.^83^

### Implications for practice

The present analysis has shown high prevalence of ED in survivors of cancer at various points and across cancer types. The aetiology of ED in survivors of cancer is multimodal with a variety of factors, including psychological and physical ones. The results should improve the visibility of this issue and allow healthcare professionals to more easily identify survivors of cancer under higher risk of ED. Moreover, it is important that clinicians be aware of the impact of ED on the quality of life and mental health of survivors of cancer, especially as sexuality and intimacy may reduce some of the psychosocial issues associated with receiving a cancer diagnosis.^19^^,^^20^ Various therapeutic modalities exist and healthcare providers should facilitate an open exchange with patients before cancer treatment and manage expectations. Primary care physicians are of great importance here given their role in follow-through during cancer care and beyond. As males are generally less prone to discuss sexual health problems in a clinical setting, clinicians should routinely and proactively ask about sexual health, recognising and acknowledging any concerns. This approach may increase patient satisfaction and improve the doctor–patient relationship.^84^
